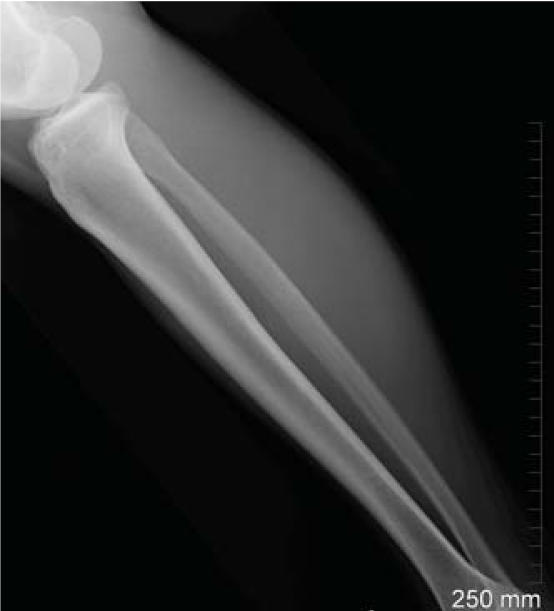# Baring Bone’s Secrets: Understanding How Lead Exposure Affects Skeletal Development

**Published:** 2007-09

**Authors:** Valerie J. Brown

Among lead’s well-known developmental health effects is stunting of skeletal growth in children. Moreover, lead is known to delay fracture healing and may contribute to osteoporosis. Yet the exact mechanism by which lead affects normal cellular functions in bone and cartilage is poorly understood. A new study reports *in vitro* and *in vivo* effects of lead on cell signaling during differentiation of embryonic stem cells destined to form bone and cartilage **[*EHP* 115:1276–1282; Zucsik et al]**.

Chondrogenesis is the process by which a mesenchymal cell—a type of stem cell already assigned to become connective tissue or blood cells—turns into a cartilage cell, or chondrocyte. As chondrocytes mature, they differentiate further into bone and more specialized types of cartilage. Because the early embryo makes a cartilage model of the skull, spine, and limbs, chondrogenesis is vital to full skeletal development.

The authors exposed mouse mesenchymal cells to lead *in vitro* and observed signaling changes in several proteins active in chondrogenesis: transforming growth factor-beta (TGF-β), bone morphogenetic protein (BMP), activating protein 1 (AP-1), and nuclear factor kappa B (NFκB). They also implanted mesenchymal cells expressing BMP-2 in the thighs of living mice. Before implantation, the mice had been exposed to lead through their drinking water.

The *in vitro* experiment revealed several dose-dependent effects. Lead stimulated chondrogenesis, influenced the regulation of chondrogenesis by BMP-2 and TGF-β, and induced expression of three genes, also mediating the genes’ regulation by BMP-2 and TGF-β. Similarly, in the *in vivo* experiment, lead was associated with a dose-dependent induction of chondrogenesis at the implantation site. Lead also inhibited AP-1 signaling and induced NFκB signaling. However, on the basis of previous research on AP-1 and NFκB with opposite findings, the authors do not believe either of these pathways is responsible for the heightened chondrogenesis observed in the current study.

TGF-β proteins are mediated by members of the Smad family of transcription activators, and Smads also affect BMP signaling in some situations. In the current study, lead inhibited BMP-2 Smad signaling while stimulating the same in TGF-β. Lead’s inhibition of BMP-2 Smad signaling “represents the most robust signaling effect identified to date in a skeletal cell type with regard to a candidate mechanism of [lead] toxicity,” the authors state. At the same time, this finding implies that lead’s influence on chondrogenesis operates independently of Smad signaling.

Given the importance of cartilage both in embryonic development and in fracture repair later in life, stimulation of chondrogenesis seems like a good thing; but if lead triggers the formation of too much cartilage at the wrong time, or prevents its further maturation into bone, this could explain lead’s crippling effects on the skeleton. And because mesenchymal cells may differentiate into a variety of cell types in addition to cartilage, lead may also affect the development of other body systems.

## Figures and Tables

**Figure:**